# REV-ERBs as regulators of circadian rhythm, neuroinflammation, and glial lipid homeostasis in Alzheimer’s disease. A narrative review

**DOI:** 10.3389/bjbs.2026.16121

**Published:** 2026-09-21

**Authors:** Fahmida Shabnam, Meenakshi Singh, Rajesh Amin, Kristine Griffett

**Affiliations:** 1 Department of Anatomy, Physiology and Pharmacology, Auburn University College of Veterinary Medicine, Auburn, AL, United States; 2 Department of Drug Discovery, Auburn University Harrison College of Pharmacy, Auburn, AL, United States

**Keywords:** Alzheimer disease, circadian rhythm, lipid homeostasis, neuroinflammation, REV-ERB

## Abstract

Alzheimer’s disease (AD) is characterized by progressive cognitive decline, amyloid-β and tau aggregation, and chronic neuroinflammation, processes that are tightly coupled to circadian and metabolic dysfunction. REV-ERBα (NR1D1) and REV-ERBβ (NR1D2) are ligand-dependent nuclear receptors that function as transcriptional repressors within the core clock and coordinate programs governing lipid metabolism, innate immunity, and redox homeostasis in the brain. In microglia, REV-ERBα restrains NF-κB signaling, complement and inflammasome activation, and lipid droplet accumulation, thereby limiting synaptic engulfment and tauopathy progression, whereas its loss drives a hyper-reactive, neurotoxic state. Astrocytic REV-ERBα exerts context-dependent effects, simultaneously constraining cytokine and nitric oxide production while tuning NAD^+^ metabolism through an NFIL3–CD38 axis with implications for tau-induced neurodegeneration. Preclinical studies demonstrate that synthetic pan-REV-ERB agonists reduce glial activation, restore NCoR/HDAC3-mediated repression, improve cognitive performance, and partially realign disrupted circadian rhythms in AD models, although current compounds are limited by suboptimal pharmacokinetics and off-target actions. In this narrative review, REV-ERBs are presented as nodal integrators of circadian, metabolic, and neuroimmune pathways in AD and as promising, yet complex, therapeutic targets, highlighting opportunities for brain-penetrant ligands, rational combinations, and chronotherapeutic dosing paradigms.

## Highlights

- REV-ERBα (NR1D1) and REV-ERBβ (NR1D2) are ligand-dependent nuclear receptors that link circadian timing, inflammation, and metabolism.
- Loss of REV-ERBα disrupts glial homeostasis, enhances NF-κB–driven inflammatory signaling, and promotes synaptic vulnerability in Alzheimer’s disease (AD).
- REV-ERB modulators attenuate microglial and astrocytic activation, restore circadian rhythmicity, and improve cognitive function in preclinical models.
- Pharmacological modulation of REV-ERBs offers a promising strategy to rebalance neuroimmune and metabolic pathways central to AD pathology.
- Integration of chronotherapeutic and combinatorial approaches could optimize the translational potential of REV-ERB–targeted therapies.

## Introduction

Alzheimer’s disease (AD) is the most prevalent cause of dementia worldwide and imposes an escalating societal and economic burden as populations age. It is characterized clinically by progressive deficits in memory and executive function and pathologically by extracellular amyloid-β (Aβ) plaques, intracellular tau neurofibrillary tangles, synaptic loss, and chronic neuroinflammation within vulnerable brain regions [1–3]. Despite substantial advances in understanding these neuropathological hallmarks, current pharmacological interventions confer at best modest symptomatic benefit and have limited impact on long-term disease progression, underscoring a critical need for mechanism-based therapeutics that target upstream drivers of neurodegeneration.

Mounting evidence indicates that circadian rhythm disruption is both highly prevalent in patients with AD and may actively exacerbate amyloid deposition, tauopathy, and neuroimmune dysfunction [2]. Core clock components regulate diverse processes including sleep–wake cycles, energy metabolism, redox homeostasis, and immune signaling, suggesting that their dysregulation could converge on key pathogenic pathways in AD [4]. Disturbances in clock-controlled neuroimmune circuits in microglia and astrocytes are increasingly recognized as central contributors to sustained neuroinflammation and synaptic vulnerability in the aging and diseased brain [5–7].

REV-ERBα (NR1D1) and REV-ERBβ (NR1D2) are heme-sensitive nuclear receptors that function as transcriptional repressors within the core circadian clockwork, where they oppose the activity of the BMAL1–CLOCK complex to shape 24-h oscillations in gene expression [8–10]. Beyond their canonical clock roles, REV-ERBs coordinate metabolic, inflammatory, and epigenetic programs by recruiting nuclear receptor co-repressor (NCoR)–HDAC3 complexes to specific DNA response elements, thereby silencing target genes involved in lipid metabolism, mitochondrial function, autophagy, and inflammasome activation. In peripheral macrophages, NR1D1 directly binds the *Nlrp3* promoter to constrain NLRP3 inflammasome activity and the maturation of interleukin-1β (IL-1β) and interleukin-18 (IL-18), positioning the REV-ERBs as gatekeepers of innate immune activation.

This review examines REV-ERBs as dynamic modulators and sensors of metabolic flux across AD-relevant cell types, rather than viewing them as binary “on/off” switches for gene transcription. We focus on the neuro-immune functions of REV-ERBα and REV-ERBβ, emphasizing their cell-type-specific actions in microglia and astrocytes. It also explores how these receptors maintain homeostasis by regulating complement and inflammasome signaling, as well as their downstream impact on synaptic integrity and neuronal survival. By synthesizing emerging mechanistic and translational data, we aim to delineate how REV-ERB dysregulation shifts the metabolic and inflammatory landscape, contributing to AD pathogenesis. Finally, we evaluate the therapeutic potential and challenges of modulating REV-ERB activity to restore circadian and neuroimmune balance in the diseased brain. Consistent with the format of a narrative review, we emphasize studies directly informing REV-ERB biology and pharmacology in AD rather than providing an exhaustive overview of all aspects of Alzheimer’s disease pathogenesis.

Rather than representing parallel pathological processes, circadian disruption, metabolic dysfunction, and neuroinflammation appear to form a self-reinforcing network that accelerates AD progression. REV-ERBs occupy a strategic position within this network because they couple clock-controlled transcriptional programs to immune and metabolic gene expression. Reduced REV-ERB activity may therefore amplify inflammatory signaling, alter glial nutrient sensing and mitochondrial homeostasis, impair protein clearance pathways, and further disrupt circadian rhythmicity. This framework suggests that REV-ERBs act as molecular integrators of disease progression rather than isolated regulators of either inflammation or circadian biology. Recent work highlighting glial nutrient sensing [11] as a determinant of AD susceptibility further supports the concept that metabolic and inflammatory pathways converge upstream of neurodegeneration [11].

## Molecular architecture and function of REV-ERBs

REV-ERBs are atypical members of the nuclear receptor superfamily that diverge structurally from classical ligand-activated receptors by lacking the carboxy-terminal activation function-2 (AF-2) helix within their ligand-binding domain (LBD), a motif normally required for coactivator recruitment and transcriptional activation (Figure 1A). As a consequence of this missing AF-2 surface, REV-ERBα and REV-ERBβ exhibit a strong preference for interaction with corepressor complexes and behave predominantly as constitutive transcriptional repressors at their response elements, often competing with transcriptionally activating ROR nuclear receptors at shared RORE sites.

**FIGURE 1:**
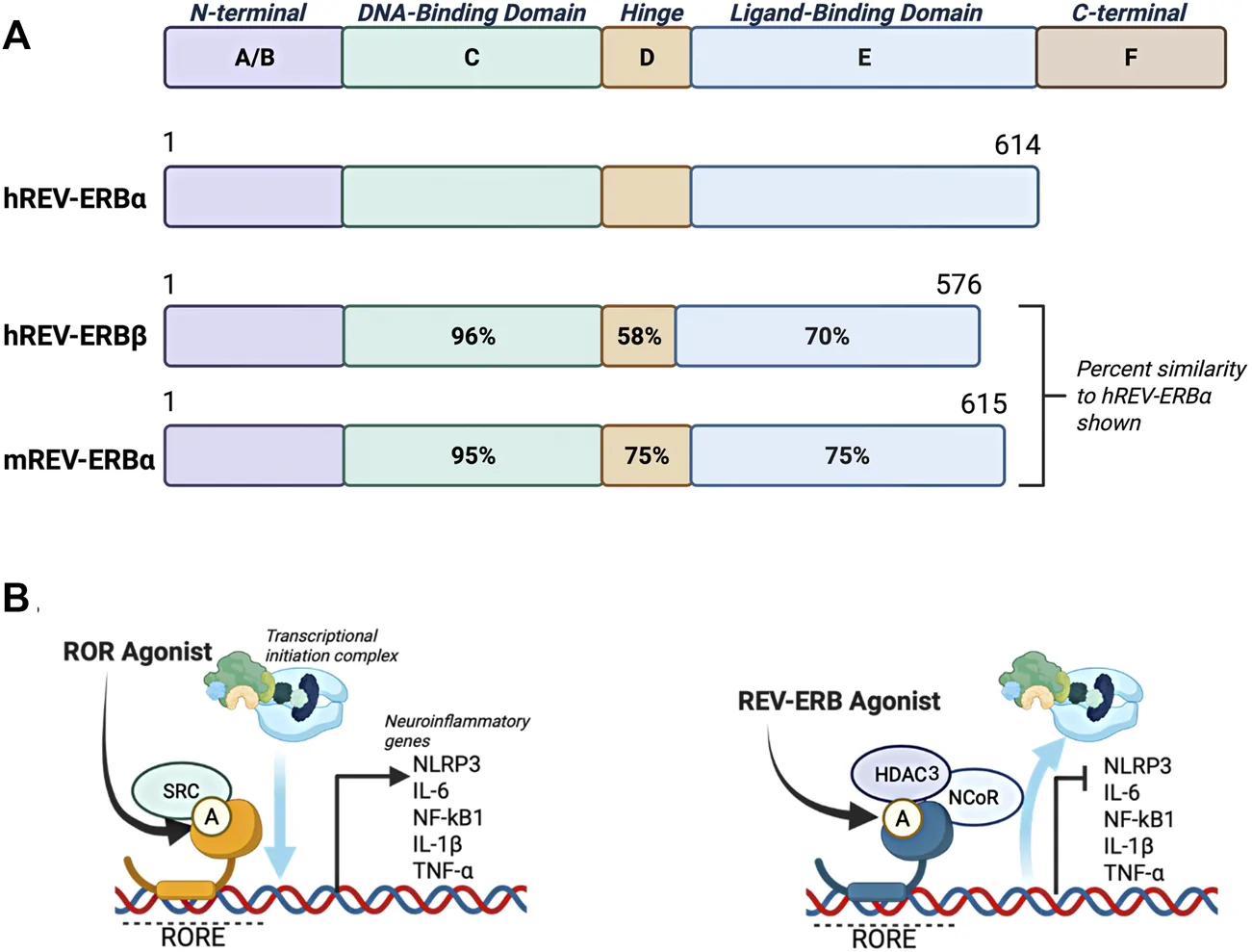
Structural and functional summary of REV-ERB nuclear receptors. **(A)** Diagram of human and mouse REV-ERBs showing conserved domains—N-terminal region, DNA-binding domain with two zinc fingers, hinge region, and ligand-binding domain. Sequence alignment indicates high similarity within the ligand-binding domain between hREV-ERBα and mREV-ERBα (75%) and between hREV-ERBα and hREV-ERBβ (70%). **(B)** Schematic comparison of ROR and REV-ERB function at RORE promoter sites. ROR agonists recruit co-activators (e.g., SRC) to promote neuroinflammatory gene expression, while REV-ERB agonists recruit co-repressors (NCoR, HDAC3), causing histone deacetylation and gene repression. Collectively, these panels illustrate that ligand-activated REV-ERBs repress ROR-induced gene activation, thereby dampening inflammatory signaling and supporting neuroimmune balance in the CNS.

Ligand engagement within the hydrophobic pocket of the LBD, most notably by endogenous heme or synthetic agonists, stabilizes a conformation of REV-ERBs that enhances high-affinity binding to the nuclear receptor corepressor (NCoR1/2) and enables the assembly of a multi-protein repression complex containing histone deacetylase 3 (HDAC3) (Figure 1B) [9]. Recruitment of NCoR–HDAC3 results in targeted deacetylation of histone tails, chromatin compaction, and exclusion of transcriptional coactivators and chromatin-looping factors, thereby enforcing durable silencing of REV-ERB target enhancers and promoters [4, 12, 13]. In parallel, at monomeric RORE sites where NCoR recruitment is weaker, REV-ERBs can still repress transcription passively by displacing RORs and limiting access of the basal transcriptional machinery [13].

Through these mechanisms, REV-ERB complexes regulate extensive transcriptional networks controlling inflammatory signaling, intermediary metabolism, and the core clock machinery [14–19]. In immune cells, REV-ERBα directly binds regulatory elements near Il-1β, interleukin-6 (Il-6), and tumor necrosis factor alpha (Tnfα), limiting their inducible expression and thereby constraining nuclear factor kappa-light-chain-enhancer of activated B cells (NF-κB)–driven inflammatory programs [20–25]. In metabolic tissues, REV-ERBs occupy enhancers of genes such as peroxisome proliferator-activated receptor gamma coactivator 1-alpha (*Pgc1α*) and Sterol regulatory element-binding protein-1 (*Srebp1*), rhythmically recruiting HDAC3 to coordinate mitochondrial biogenesis, fatty acid oxidation, and lipogenesis with circadian nutrient availability [14, 21, 25]. At the level of the clock, REV-ERBα/β form a negative feedback arm by repressing *Bmal1* and other BMAL1-CLOCK targets, creating interlocked loops that couple cellular metabolic state to 24-h oscillations in gene expression [16, 17, 19, 25].

The convergence of inflammatory, metabolic, and circadian transcriptional control positions REV-ERBs as molecular integrators of “immune–metabolic timing,” translating oscillations in heme and redox state into coordinated changes in chromatin accessibility and gene output across diverse cell types [15, 19, 23, 26]. In the nervous system, where precise temporal regulation of energy use and immune surveillance is essential for synaptic maintenance, disruption of REV-ERB–NCoR–HDAC3 signaling is poised to amplify maladaptive cytokine production, alter lipid handling, and disturb clock gene coherence, thereby accelerating vulnerability to neurodegenerative processes [23].

Although REV-ERBα and REV-ERBβ share substantial sequence homology and bind similar response elements, accumulating evidence suggests that they are not completely redundant. REV-ERBα exhibits stronger circadian oscillation and is highly enriched in metabolically active tissues and immune cell populations, whereas REV-ERBβ displays broader constitutive expression and may provide transcriptional stability when REV-ERBα oscillations are low. Within the CNS, both isoforms are detected in neurons and glia, but most mechanistic studies in AD have focused on NR1D1 because genetic manipulation of REV-ERBα produces robust effects on neuroinflammation, synaptic pruning, lipid metabolism, and tau pathology. In contrast, comparatively little is known regarding the specific contribution of REV-ERBβ to AD pathogenesis, representing an important knowledge gap for future investigation [14, 21, 27].

REV-ERBs function within an interconnected transcriptional network composed of BMAL1, CLOCK, PER, CRY, ROR, DBP, and additional clock-controlled factors. BMAL1-CLOCK heterodimers activate transcription of Per, Cry, Rev-erb, and Ror genes through E-box elements. PER and CRY proteins subsequently repress BMAL1-CLOCK activity, while REV-ERBs and RORs form a secondary stabilizing feedback loop by competing for RORE sites within target promoters. REV-ERBs suppress Bmal1 transcription through recruitment of NCoR-HDAC3 complexes, whereas RORs activate Bmal1 expression. Through these opposing actions, REV-ERBs synchronize inflammatory, metabolic, and behavioral rhythms with environmental light-dark cycles. Given that altered BMAL1, PER, CRY, and REV-ERB rhythmicity have all been reported in AD, disruption of one component may propagate throughout the entire circadian system and amplify neurodegenerative processes [13, 15, 18, 19].

## CNS expression and cell-type specificity

REV-ERBs are broadly expressed across peripheral and central tissues, consistent with their role as systemic regulators of circadian and metabolic physiology, but transcriptomic and proteomic analyses reveal significantly reduced levels of REV-ERBα in AD brains, particularly within vulnerable CNS regions such as the frontal cortex, hippocampus, and suprachiasmatic nucleus (SCN) [25, 26, 28]. In both human postmortem tissue and AD mouse models, these reductions coincide with disrupted clock gene rhythmicity and impaired coupling between the SCN and downstream oscillators, suggesting that loss of REV-ERBα tone contributes to the global circadian disorganization characteristic of AD [28–32].

Within the healthy brain, *NR1D1* and *NR1D2* are detected in neurons, astrocytes, and microglia, and their expression exhibits robust day-night oscillations that mirror rhythmic changes in cellular metabolism and inflammatory responsiveness [9, 10, 33–37]. These diurnal patterns are particularly prominent in SCN neurons but are also evident in hippocampal and cortical circuits as well as in glial populations, where clock-controlled REV-ERB activity constrains basal cytokine production and shapes time-of-day differences in synaptic pruning and immune surveillance [34, 35, 38, 39].

Single-cell and single-nucleus RNA-seq datasets from human AD cortex and hippocampus now provide cellular resolution to these alterations, demonstrating a consistent downregulation of NR1D1 transcripts in disease-associated microglia (DAM) and subsets of reactive astrocytes, relative to homeostatic counterparts in control brains [33, 34]. DAM and reactive astrocyte clusters with reduced *NR1D1* show enrichment of pro-inflammatory gene modules and complement and chemokine signatures, implicating loss of REV-ERBα repression as a key step in the transition from physiological to pathological glial states [33, 34, 40].

Experimental data from other neurodegenerative contexts support a causal link between decreased *NR1D1* and maladaptive glial activation: *NR1D1* downregulation in astrocytes induces a pro-inflammatory, neurotoxic phenotype, while perturbation of REV-ERBα/β signaling in microglia alters their activation and phagocytic capacity [41–43]. Together, these observations suggest that diminished REV-ERB expression and rhythmicity weaken intrinsic brakes on glial inflammatory programs, thereby amplifying neuroinflammatory tone and lowering the threshold for neuronal injury in AD and related neurodegenerative diseases.

Human transcriptomic studies increasingly support clinical relevance of REV-ERB dysregulation in AD. Single-cell datasets demonstrate reduced NR1D1 expression in disease-associated microglia and reactive astrocytes, while broader transcriptomic analyses reveal altered expression of REV-ERB-regulated inflammatory and metabolic pathways in vulnerable cortical regions [44]. Emerging biomarker frameworks such as ATX(N) emphasize incorporation of inflammatory, metabolic, vascular, and synaptic markers alongside amyloid and tau measures, suggesting that REV-ERB-regulated biological processes may contribute to clinically measurable disease signatures [44]. At present, direct measurements of NR1D1 or NR1D2 proteins in CSF or blood remain limited, and most evidence linking REV-ERB pathways to human disease derives from transcriptomic datasets and postmortem tissue analyses rather than validated fluid biomarkers.

## REV-ERB modulators and pharmacology

The first-generation synthetic agonist GSK4112, identified through fluorescence resonance energy transfer assays, established proof-of-concept that small molecules can ligand-dependently enhance REV-ERB–NCoR interactions and repress canonical target genes such as *Bmal1*, but its low potency, poor metabolic stability, and negligible systemic exposure have limited its use largely to *in vitro* studies and acute *ex vivo* assays [45]. Second-generation benzimidazole derivatives SR9009 and SR9011 were subsequently developed to improve drug-like properties, exhibiting greater bioavailability, central nervous system penetration, and *in vivo* efficacy in models of metabolic syndrome and neuroinflammation (Figure 2) [25, 45]. In microglia and macrophages, these agonists attenuate the production of pro-inflammatory cytokines, suppress NLRP3 inflammasome activation, and shift cellular metabolism toward a less oxidative, lower-ATP state, thereby linking pharmacologic pan-REV-ERB activation to coordinated immunometabolic reprogramming [25, 46, 47]. Nonetheless, SR9009 and SR9011 display relatively short plasma half-lives, off-target effects at higher concentrations, and modest receptor affinity, which together constrain their translational potential and complicate interpretation of long-term *in vivo* studies [25].

**FIGURE 2:**
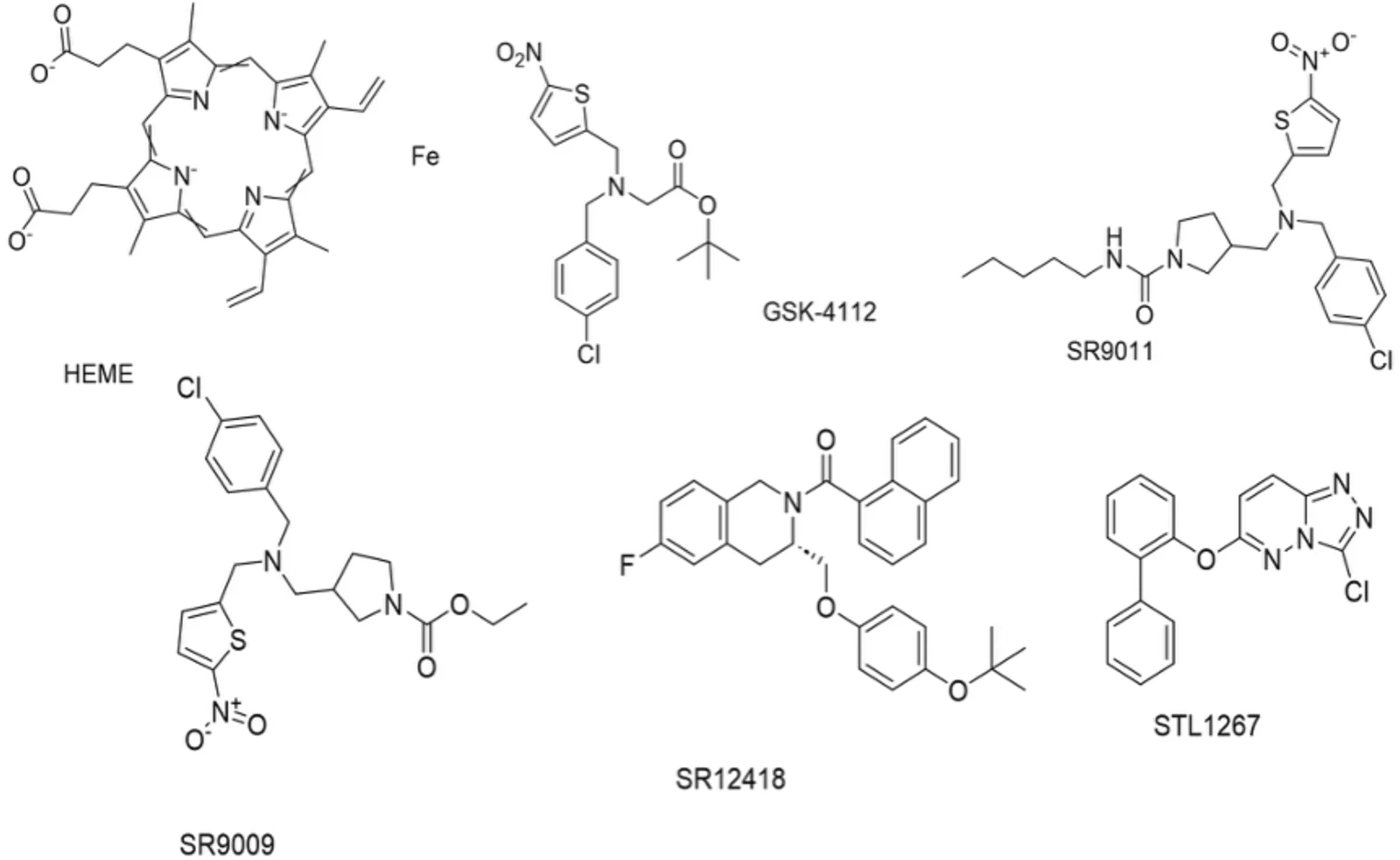
Representative chemical structures of the endogenous ligand heme and synthetic REV-ERB agonists.

In contrast, the competitive antagonist SR8278 disrupts ligand-dependent REV-ERB-corepressor binding, derepressing REV-ERB target genes and, in some tissues such as skeletal muscle, enabling REV-ERB to tether to other transcription factors and modulate non-canonical signaling pathways [48]. In microglial contexts, short-term SR8278 treatment enhances Aβ clearance by upregulating phagocytic receptors including TREM2 and P2Y12 and by boosting microglial chemotaxis, suggesting that transient release of REV-ERB-mediated repression can acutely favor plaque-engulfing phenotypes [49]. However, chronic or high-dose antagonist exposure has been associated with exacerbated astrogliosis, increased pro-inflammatory gene expression, and tissue remodeling in other organs, indicating that sustained blockade of REV-ERB’s repressive function can drive maladaptive gliosis and inflammation [49]. These bidirectional outcomes highlight that both the magnitude and temporal pattern of REV-ERBα/β modulation, as well as cell-type-specific receptor expression and chromatin context, are critical determinants of whether pharmacologic interventions ultimately promote neuroprotection or neurotoxicity.

More recently, the high-affinity pan-agonist STL1267 has emerged as a next-generation tool compound with markedly improved potency and pharmacokinetic properties relative to SR9009, including submicromolar binding to REV-ERBα, enhanced metabolic stability, and superior *in vivo* exposure [50]. Structural studies reveal that STL1267 occupies a deep hydrophobic cavity within the REV-ERBα LBD and induces a distinct conformational ensemble that optimizes co-repressor engagement, providing a molecular explanation for its enhanced efficacy (Figure 3) [27, 50]. Functionally, STL1267 robustly suppresses macrophage NLRP3 inflammasome activation and downstream secretion of IL-1β, IL-18, and IL-6 in inflammatory pain models, positioning REV-ERB activation as an attractive strategy to target cytokine and inflammasome pathways that are also central to AD-associated neuroinflammation [51].

**FIGURE 3:**
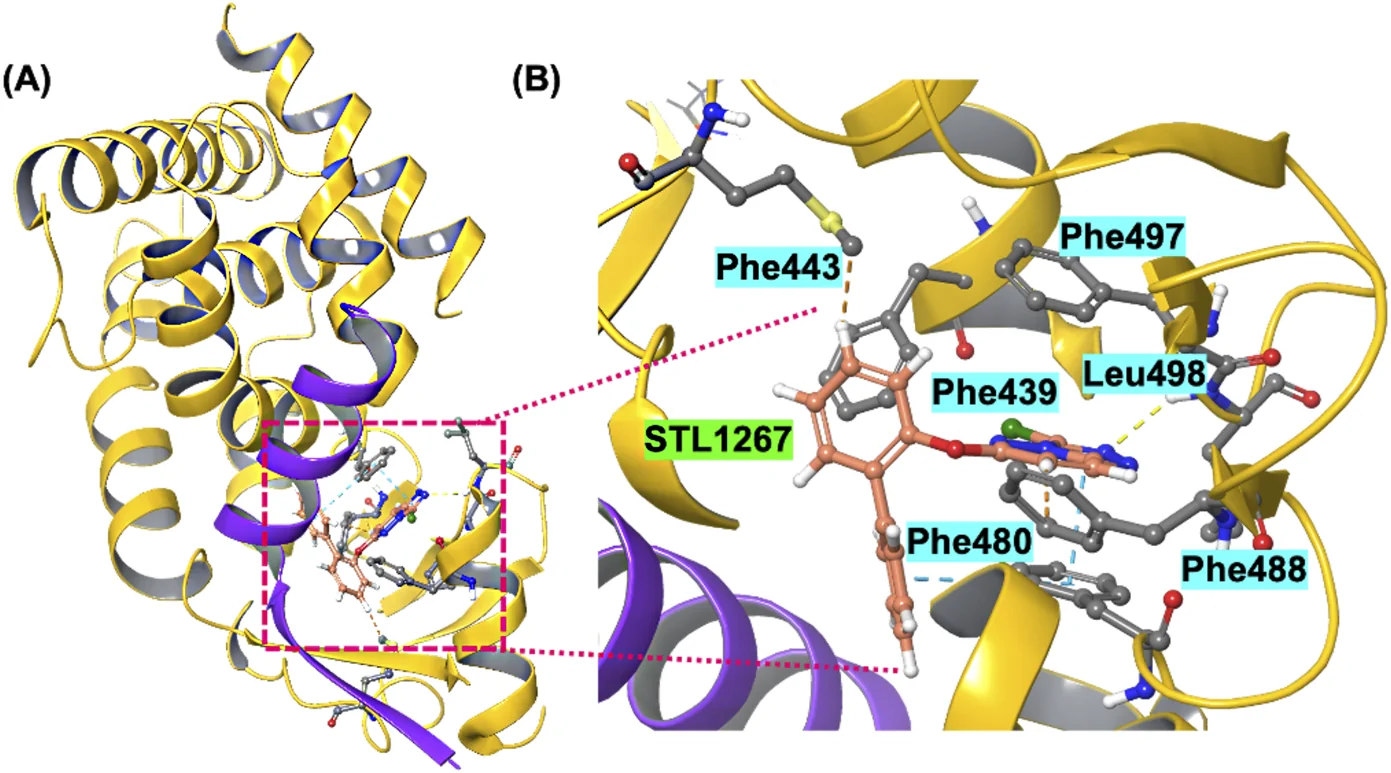
Crystal structure of STL1267 bound to the ligand binding domain of REV-ERBα. **(A)** STL1267 binds directly to the ligand-binding domain (LBD) of REV-ERB, leading to the recruitment of corepressor proteins (NCoRD1) and the regulation of downstream target genes, such as the repression of BMAL1 gene expression. This induced beta-sheet formation is critical to explain how STL1267 recruits corepressors, a mechanism that is distinct from the one seen with heme. The binding pocket is different from the one occupied by the endogenous ligand, heme, which binds in a region closer to helix-11 and the corepressor binding surface. REV-ERBα is shown in yellow ribbons and NCoR ID1 in purple ribbons. The ligand is shown stick representation in the ligand binding domain (orange color). STL1267 binds to a hydrophobic pocket, which is deep within the ligand-binding domain (LBD) of REV-ERBα. This pocket is located closer to helix-3 compared to the heme binding site Key interactions of STL1267 with amino acids within the nuclear receptor LBD are designated. **(B)** Zoom-in-view of STL1267 binding within the ligand binding pocket of REV-ERBα. The ligand is illustrated as orange stick representation and the protein amino side chains are demonstrated by yellow stick representation. Hydrophobic amino acids stabilize STL1267 in the ligand binding pocket of REV-ERBα. Note key amino acid interactions of STL1267 with REV-ERBα.

Structure-activity relationship analyses around STL1267 and related scaffolds have identified a cluster of residues within the LBD, particularly phenylalanine 484, 488, and 497, together with leucine 498, as critical determinants of agonist binding and transactivation, forming a hydrophobic microenvironment that cradles the ligand (Figure 3A) [50, 52]. The close packing of aromatic and aliphatic side chains around these residues allows small molecules to establish extensive van der Waals and π–π interactions, stabilizing the agonist-bound conformation that favors helix positioning for productive NCoR/HDAC3 recruitment (Figure 3B). Exploiting this hydrophobic hotspot through rational design and computational docking is central to current efforts to generate brain-penetrant, isoform-selective REV-ERB agonists with optimized potency, selectivity, and safety profiles, thereby advancing REV-ERB ligands toward clinical application in AD and other neuroinflammatory disorders.

## REV-ERBs in glial crosstalk and neuroinflammation

AD pathology is increasingly recognized as an emergent property of bidirectional communication between microglia and astrocytes, which together coordinate neuronal energy supply, synaptic remodeling, and responses to injury, and whose cross-talk is under circadian and transcriptional control by REV-ERBs [35, 53, 54]. Microglia, the resident innate immune cells of the CNS, sense Aβ plaques and pathological tau species via pattern-recognition receptors and shift from a surveillant, ramified morphology to an activated phenotype characterized by production of reactive oxygen and nitrogen species and release of pro-inflammatory mediators. Among these, TNF-α, IL-1β, and IL-6 constitute a core cytokine triad that drives the ensuing neuroinflammatory cascade, amplifying synaptic dysfunction, promoting additional Aβ deposition, and facilitating tau hyperphosphorylation and spread [2]. ​

Astrocytes normally support synaptic transmission, regulate extracellular glutamate and potassium, and couple neuronal activity to local energy and blood flow, thereby preserving metabolic homeostasis. In AD, however, astrocytes undergo reactive astrogliosis and acquire context-dependent A1/A2-like states marked by upregulation of pro-inflammatory cytokines, complement components, and nitric oxide, which contribute to excitotoxicity, synapse loss, and disruption of blood–brain barrier (BBB) integrity [42]. Reactive astrocytes not only fail to clear Aβ efficiently but can themselves produce Aβ under inflammatory conditions, further fueling plaque dynamics and vascular dysfunction [42].

Reciprocal signaling between activated microglia and astrocytes establishes a self-reinforcing loop in which microglial cytokines, including TNF-α and IL-1 family members, drive astrocytes toward neurotoxic, complement-secreting phenotypes, while astrocyte-derived factors in turn perpetuate microglial activation and impair their phagocytic handling of Aβ and tau [29–32, 36]. This vicious cycle heightens oxidative stress, perturbs neuronal bioenergetics, and accelerates progressive neurodegeneration in vulnerable cortical and hippocampal circuits. Within this network, REV-ERBα acts as a controlled transcriptional brake: genetic deletion or pharmacologic inhibition of REV-ERBα in mice leads to spontaneous microglial and astrocyte activation, enhanced NF-κB signaling, and increased complement C3 expression, whereas pan-REV-ERB agonists dampen glial inflammatory tone and improve neuronal outcomes [33, 34, 42, 55–57]. These findings support a model in which intact REV-ERB signaling restrains excessive microglia–astrocyte reactivity, preserving the balance between protective immune surveillance and pathological neuroinflammation in AD.

## Microglial REV-ERBα in inflammatory regulation

REV-ERBα is expressed in microglia and contributes importantly to the regulation of inflammatory transcriptional programs. In global Nr1d1 knockout (RKO) mice, the loss of this repression leads to spontaneous hippocampal microgliosis and secondary astrogliosis. This is accompanied by enhanced NF-κB p65 nuclear accumulation and the upregulation of pro-inflammatory cytokines and complement factors, including *Il-1β*, *Tnf*α, and *C1q*, which together produce neuronal dysfunction that phenocopies early stages of AD-like pathology. Beyond cytokines, *Nr1d1* deletion disrupts the circadian control of complement gene expression, producing exaggerated diurnal swings and an overall elevation of chemokines such as *Ccl2* and *Cxcl10*, complement components C3 and C4b, and inflammasome-associated transcripts like *Nlrp3* and *Casp1*. These changes collectively favor excessive synaptic tagging and microglial phagocytosis. Consistent with the broader importance of inflammasome signaling in AD, recent studies have identified USP16-dependent regulation of IFI16 and TLR4/NLRP3 pathways as additional mediators of Aβ1-42-induced inflammatory responses. Suppression of IFI16 reduced cytokine production and attenuated neuronal injury in glial-neuronal co-culture systems, reinforcing the role of innate immune signaling as a central driver of AD-associated neurotoxicity [58].

Tauopathy models provide further evidence that microglial REV-ERBα acts as a critical node linking inflammatory tone, lipid metabolism, and protein aggregate clearance. In PS19 mice expressing mutant human P301S tau, microglia-specific *Nr1d1* deletion exacerbates tau aggregation and neurofibrillary tangle burden. Notably, these effects are sex-specific, occurring only in male mice, while female mice show no exacerbation of tau pathology. In affected males, the loss of REV-ERBα increases the expression of inflammatory cytokines and drives the accumulation of lipid droplets within microglia. This “clogged” metabolic state impairs their phagocytic capacity for pathological tau species. Pharmacologic or genetic blockade of lipid droplet formation partially restores tau uptake in these microglia, indicating that REV-ERBα-dependent regulation of lipid homeostasis is integral to the efficient clearance of tau aggregates [38, 55]. This sex-dependence has important implications for therapeutic development and clinical translation.

Strikingly, the depletion of microglia in RKO mice normalizes many of the behavioral and cognitive abnormalities and attenuates neuroinflammatory signatures [38]. This demonstrates that hyperactivated microglia are the principal effectors of the neuropathology that emerges when REV-ERBα signaling is lost. Together, these data support a model in which REV-ERBα enforces microglial homeostasis by coordinating the circadian repression of NF-κB, complement, and inflammasome pathways and by maintaining lipid metabolic balance. In this context, REV-ERBα functions as a brake on inflammatory activation; its presence prevents microglia from entering a chronically reactive, neurotoxic state, while its activation via agonism is required to maintain the microglial capacity for proteostasis.

Importantly, not all proposed effects of REV-ERBα have been demonstrated through direct promoter occupancy or loss-of-function experiments. Direct evidence supports regulation of NF-κB signaling, complement expression, microglial synaptic phagocytosis, lipid droplet accumulation, and tau pathology. In contrast, links between REV-ERBα and broader inflammasome networks, oxidative stress pathways, and certain aspects of amyloid pathology remain largely mechanistic inferences derived from overlapping transcriptional programs or observations in peripheral immune cells. Distinguishing experimentally validated functions from extrapolated models will be important as the field moves toward therapeutic translation [33–35, 38].

## Astrocytic REV-ERBα and metabolic adaptation

Astrocyte-specific *Nr1d1* deletion elicits a noncanonical response compared with the detrimental effects of microglial REV-ERBα loss. In the astrocyte compartment, the absence of this receptor induces an increase in bulk brain and astrocytic NAD^+^ levels [29, 33, 37, 42, 59, 60]. This occurs via an NFIL3-CD38 signaling axis where REV-ERBα deficiency leads to the derepression of NFIL3, which subsequently limits CD38-dependent NAD^+^ consumption and preserves intracellular NAD^+^ pools [31, 42, 55]. In P301S tauopathy models, this astrocytic REV-ERBα deficiency attenuates tau aggregation, neuroinflammation, and neuronal loss [31, 42, 55]. These findings indicate that reduced astrocytic REV-ERBα activity can paradoxically confer neuroprotection by enhancing NAD^+^-dependent stress resilience and proteostasis. In this specific metabolic context, REV-ERB antagonism, rather than activation appears beneficial by boosting NAD^+^ availability and supporting mitochondrial health.

In contrast, the reduction of *NR1D1* in primary astrocyte cultures or human iPSC-derived astrocytes drives a robust pro-inflammatory shift. This is characterized by the upregulation of *IL-6*, *NOS2*, and *NF-κB* reporter activity, alongside the acquisition of a neurotoxic phenotype that compromises the survival of cocultured neurons [31, 42]. These observations suggest that astrocytic REV-ERBα functions as a dual-purpose regulator: it simultaneously restrains inflammatory gene expression and tunes redox and NAD^+^ metabolism.

The net outcome of REV-ERBα modulation in astrocytes appears to depend on the disease stage, the specific injury context, and the balance between its roles in immune repression versus metabolic control. Thus, astrocytic REV-ERBα coordinates metabolic adaptation with inflammatory reactivity, positioning this clock receptor as a context-dependent regulator of astrocyte-neuron interactions. This divergence from microglial signaling highlights that a “one-size-fits-all” therapeutic approach to REV-ERB may be counterproductive; while microglia may require agonism to maintain phagocytic clearance, astrocytes may benefit from targeted antagonism to preserve energy homeostasis.

Recent reviews of glial nutrient sensing further support the concept that microglia and astrocytes integrate metabolic cues with inflammatory responses during AD progression. Alterations in nutrient availability and utilization can reshape glial energy metabolism, cytokine secretion, and phagocytic capacity, thereby influencing downstream neurodegenerative outcomes [11].

## Pharmacological activation in preclinical AD models

Activation of REV-ERBs confers broad neuroprotective effects across multiple experimental paradigms, including amyloid- and tau-driven models of AD. In APP/PS1 mice, systemic administration of REV-ERB agonists such as SR9009 or SR9011 reduces microgliosis and astrogliosis, attenuates NF-κB-dependent inflammatory gene programs, and restores performance in hippocampal-dependent memory tasks, indicating that modulation of clock-controlled transcription can reverse both cellular and behavioral indices of pathology [33, 34, 38, 54, 55]. These benefits extend to models of LPS-induced neuroinflammation and traumatic brain injury, suggesting that REV-ERB activation broadly dampens CNS innate immune activation and preserves neuronal integrity in inflammatory contexts.

While the traditional view of REV-ERB emphasizes its role as a transcriptional repressor, emerging evidence in neurodegeneration suggests it functions as a modular sensor of cellular state rather than a static “off-switch.” This is particularly evident in the regulation of autophagy, where REV-ERB activation has been reported to suppress autophagic flux depending on the pathological context and the specific metabolic needs of the cell. This ability to sense cellular needs implies that REV-ERB coordinates autophagic machinery in response to real-time metabolic flux and protein aggregate burden [61–65]. In some contexts, REV-ERB may repress specific autophagy-related genes (ATGs) to prevent excessive self-digestion or to synchronize degradation with circadian cycles. This has been reported in numerous studies in the context of cancer biology under extremely hypoxic conditions, which likely alter the availability of heme and the interaction of heme within the ligand binding domain of REV-ERB [66, 67]. Conversely, in the presence of heavy amyloid or tau accumulation, REV-ERB may act as a homeostatic sensor that facilitates the derepression of clearance pathways or modulates upstream signaling nodes, such as the NFIL3-CD38-NAD^+^ axis in astrocytes or lipid droplet dynamics in microglia, to indirectly enhance autophagic efficiency [38, 55]. Rather than a binary controller of gene expression, REV-ERB likely serves as a physiological rheostat, tuning the autophagic response to maintain cellular proteostasis and energy balance amidst the fluctuating stresses of the diseased brain [61–65].

Despite these promising preclinical data, currently available agonists suffer from rapid systemic clearance, limited oral bioavailability, and off-target activities that complicate interpretation of long-term studies and pose challenges for clinical development. Optimization efforts now focus on designing next-generation ligands, such as STL1267 and related scaffolds, with improved metabolic stability, CNS penetration, and isoform selectivity, alongside formulation strategies that sustain target engagement over the circadian cycle. Moving forward, elucidating cell-type-specific pharmacodynamics in microglia, astrocytes, and neurons, and defining exposure-response relationships for both beneficial and adverse effects, will be essential to translate REV-ERB activation into a viable therapeutic approach for AD [51].

Interpretation of cognitive improvements warrants caution because REV-ERB agonists alter both neuroimmune signaling and circadian organization. Improvements in learning and memory may therefore arise from multiple mechanisms, including reduced inflammatory burden, improved sleep architecture, restoration of circadian coherence, or combinations thereof. Most available studies do not experimentally separate these variables, making it difficult to determine the relative contribution of direct neuroimmune modulation versus secondary behavioral effects.

## Therapeutic perspectives and chronopharmacology

Given the intertwined nature of circadian, metabolic, and inflammatory dysfunction in AD, targeting REV-ERBs offers a uniquely integrative strategy to recalibrate multiple pathological axes simultaneously rather than focusing on a single lesion such as plaques or tangles. REV-ERB agonism can, in principle, suppress NF-κB- and NLRP3-driven neuroinflammation, normalize glial lipid handling, and restore clock-controlled transcriptional rhythms, thereby improving proteostasis, synaptic function, and vascular-metabolic coupling within the same therapeutic framework. Building on this pleiotropy, combinatorial approaches that pair REV-ERB agonists with NLRP3 inhibitors, anti-amyloid antibodies, tau-directed biologics, or GLP-1 receptor agonists may yield additive or synergistic benefits by concurrently reducing innate immune activation, enhancing aggregate clearance, and improving systemic metabolic status, all of which influence AD trajectory [56, 57, 68, 69].

A critical question for such strategies is how best to exploit the intrinsic rhythmicity of REV-ERB signaling. Circadian disruption and aberrant expression or phosphorylation of core clock proteins (BMAL1, CLOCK, PER/CRY, REV-ERBα) are now recognized as early and pervasive features of AD, Parkinson’s disease, and related proteinopathies, and experimental perturbation of these factors can directly modulate microglial and astrocytic activation, Aβ and tau aggregation, and neuronal susceptibility to oxidative and excitotoxic stress [8–10]. These observations raise the possibility that aligning drug administration with endogenous REV-ERB oscillations, delivering agonists around predicted peaks of target expression or activity, could maximize on-target repression of inflammatory programs while limiting off-target effects on essential clock outputs in peripheral tissues.

Preclinical studies already provide proof-of-principle for such chronotherapeutic REV-ERB modulation. In rodent models, restricting dosing of REV-ERB ligands or other anti-inflammatory agents to specific circadian phases improves restoration of clock gene rhythms in hippocampus and cortex, enhances diurnal variation in glial activation markers, and more effectively reduces cytokine levels and complement activation than phase-mismatched schedules [68]. Time-restricted administration also appears to better preserve sleep-wake architecture and cognitive performance, suggesting that respecting and reinforcing circadian structure may be as important as achieving a given cumulative exposure [68]. Translating these insights will require defining cell-type-specific pharmacodynamics of emerging, brain-penetrant REV-ERB ligands across the 24-h cycle in humans and integrating actigraphy, melatonin rhythms, and fluid biomarkers of neuroinflammation into early-phase trials to identify optimal dosing windows and combinations.

Long-term systemic activation of REV-ERBs may produce adverse consequences because these receptors regulate lipid metabolism, glucose homeostasis, skeletal muscle energetics, immune responses, and cardiovascular physiology. Interventions that are beneficial within microglia could therefore generate unwanted metabolic or circadian effects in peripheral tissues. The challenge may be particularly relevant for elderly AD patients, who frequently exhibit multimorbidity, metabolic disease, and altered sleep-wake rhythms. Development of brain-penetrant compounds, cell-selective targeting approaches, and chronopharmacological dosing schedules may help mitigate these risks [14, 21, 25, 27].

Available evidence suggests that REV-ERBα activity is unlikely to be uniformly beneficial across all disease stages. Early disease may benefit from enhanced microglial phagocytic function to promote Aβ clearance, whereas excessive repression of inflammatory programs during late-stage disease could impair adaptive immune responses. Furthermore, sex-dependent effects have been observed in tauopathy models, and astrocytic responses differ markedly from those observed in microglia. Collectively, these findings argue for stage-specific and cell-type-specific therapeutic strategies [38, 49, 55].

## Perspectives and conclusion

The evidence synthesized here positions REV-ERBs as modular, homeostatic sensors at the intersection of circadian timing, metabolic flux, and neuroimmune function (Figure 4). By integrating heme and redox status with transcriptional programs that govern complement activation, inflammasome signaling, lipid handling, and core clock gene expression, REV-ERBα and REV-ERBβ act as physiological rheostats that shape glial states across the 24-h cycle. Dysregulation of this system, through reduced NR1D1/NR1D2 expression in vulnerable brain regions, loss of rhythmicity in glial clock gene networks, and context-dependent changes in astrocytic NAD^+^ metabolism, is a recurrent feature of Alzheimer’s disease (AD) and related tauopathies. These findings suggest that REV-ERBs are active determinants of whether glial responses remain protective or shift into chronic, neurotoxic inflammation (Table 1).

**TABLE 1:** Cell-type-specific REV-ERB functions in AD.

Cell type	Primary regulatory axis	Effect of REV-ERB loss	Therapeutic goal
Microglia	Lipid metabolism & phagocytosis	Increased lipid droplets; impaired tau clearance	Agonism (activation)
Astrocytes	NFIL3-CD38-NAD⁺ metabolic axis	Preserved NAD⁺ levels; neuroprotection	Antagonism (inhibition)
Neurons	Circadian transcriptional feedback	Loss of homeostatic synaptic genes	Stabilization

**FIGURE 4:**
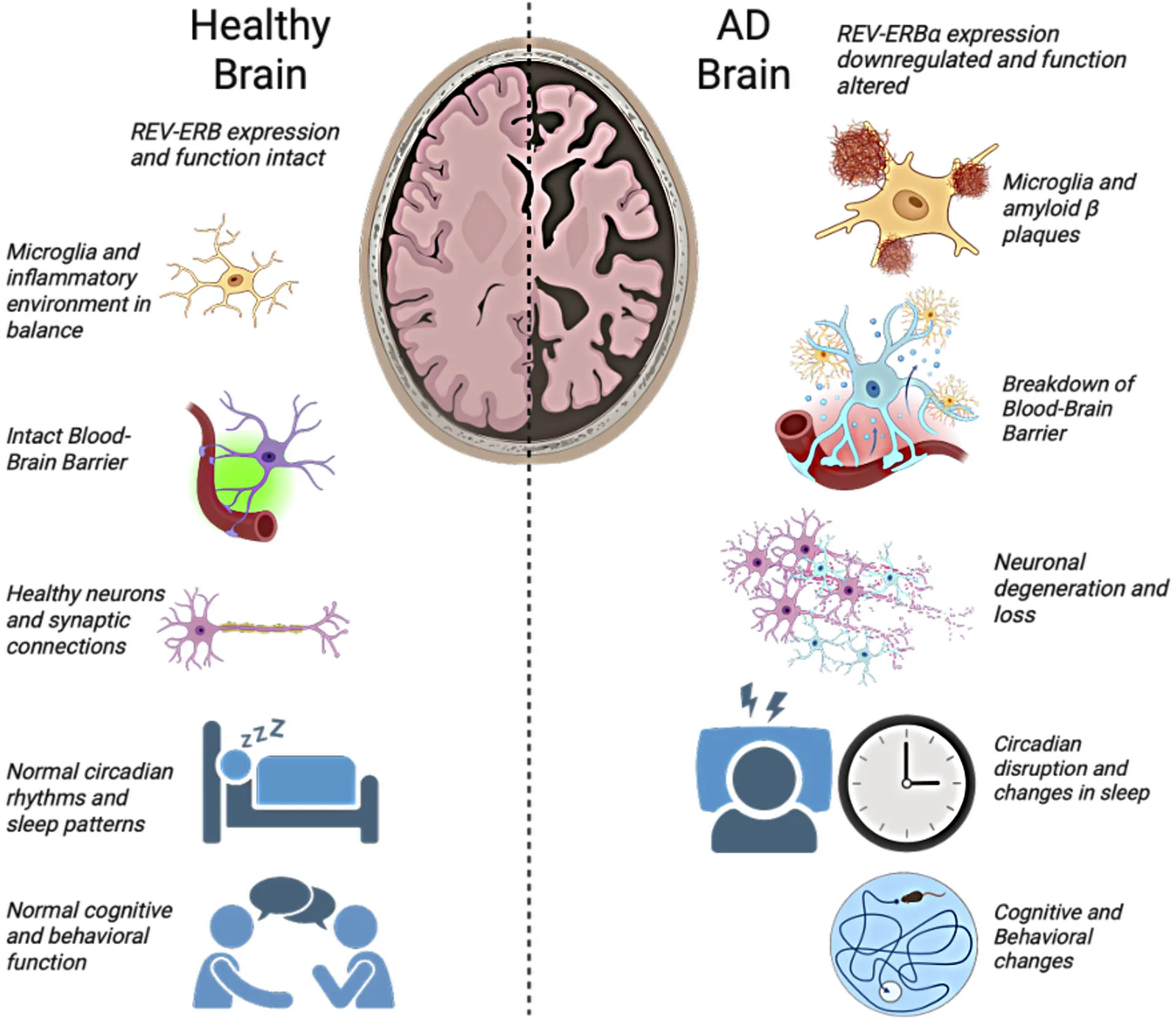
REV-ERB-Dependent Neurobiology in Health and Alzheimer’s Disease. Left (Healthy Brain): REV-ERB expression supports balanced microglial activity, blood–brain barrier integrity, neuronal connectivity, and synchronized circadian and sleep rhythms, sustaining normal cognition and behavior. Right (AD Brain): In Alzheimer’s disease, reduced REV-ERB expression and activity promote microglial activation, amyloid-β accumulation, blood–brain barrier disruption, and neurodegeneration, accompanied by circadian and sleep disturbances and cognitive impairment. The figure highlights how REV-ERB dysfunction disrupts neuroimmune and metabolic control, driving neurodegenerative progression.

At the microglial level, genetic and pharmacological data show that REV-ERBα enforces homeostasis by repressing NF-κB-driven cytokine cascades, complement components, and NLRP3 inflammasome genes. It influences lipid droplet dynamics and may contribute to processes involved in proteostasis and aggregate clearance. The loss of Rev-erbα transforms microglia into chronically reactive cells that overproduce cytokines, accumulate neutral lipids, and fail to clear Aβ and tau, which accelerates synaptic loss and behavioral decline in tauopathy models. Since microglial depletion can rescue phenotypes in global *Nr1d1* knockout mice, dysfunction in this single glial compartment appears sufficient to drive substantial neuropathology.

Astrocytic REV-ERBα plays a more nuanced role that depends on the surrounding environment. While it typically constrains IL-6 and nitric oxide production to limit neurotoxic reactive states, its loss can also increase NAD^+^ availability through NFIL3–CD38 signaling. This specific pathway provides partial protection against tau-induced neurodegeneration. Such divergent outcomes emphasize that cellular context, disease stage, and the metabolic environment are critical when interpreting REV-ERB function. These results suggest that therapeutic success will rely on cell-type-selective modulation or strategies that target specific disease stages.

Pharmacological studies provide a translational perspective but also highlight current limitations. First- and second-generation agonists, including GSK4112, SR9009, and SR9011, show that increasing REV-ERB activity reduces glial activation, dampens inflammasome signaling, and improves memory in amyloid and inflammatory models. Newer ligands like STL1267 offer better potency and help clarify the structural details of agonist binding and co-repressor recruitment. However, many compounds have short half-lives, poor brain penetration, and off-target effects. Antagonists like SR8278 also show that temporary inhibition of REV-ERBs might help Aβ phagocytosis in certain microglial states, while chronic blockade makes astrogliosis and neuroinflammation worse.

The rhythmic nature of REV-ERB signaling adds another layer of complexity. Circadian disruption happens early in AD, and core clock components like REV-ERBα directly affect neuroinflammation and neuronal vulnerability. This suggests that targeting REV-ERBs should involve specific timing. Aligning drug delivery with peaks of receptor expression or windows of high glial responsiveness could improve results while avoiding interference with essential clock functions in the body. While preclinical studies on time-restricted feeding and phase-specific drug administration are promising, there is little data on how this applies to humans.

Future research should prioritize cell-type and time-resolved mapping of REV-ERB chromatin occupancy in human and experimental AD. This is necessary to define exactly when and where these receptors are helpful or harmful. Next-generation ligands also need better brain penetration and isoform selectivity to allow for precise dosing regimens. Integrating REV-ERB-targeted treatments with existing AD therapies, such as anti-amyloid antibodies or NLRP3 inhibitors, could lead to combination strategies that improve aggregate clearance while calming the neuroimmune system. Finally, early clinical studies should track circadian biomarkers like sleep-wake metrics and melatonin rhythms alongside standard neurodegeneration markers.

Emerging ATX(N)-based biomarker frameworks increasingly recognize inflammatory, vascular, metabolic, and synaptic pathways as clinically relevant components of AD biology beyond amyloid and tau alone. Future studies integrating NR1D1/NR1D2 expression, circadian biomarkers, blood-based inflammatory markers, and transcriptomic signatures may help determine whether REV-ERB pathway dysfunction can serve as a clinically informative biomarker of disease progression [44].

In conclusion, REV-ERBs sit at the crossroads of circadian biology, metabolism, and neuroimmunity, making them strong candidates for intervention in AD. The literature suggests that well-timed, cell-type-aware modulation of REV-ERB signaling can restore glial homeostasis and improve cognitive outcomes. However, the different roles of REV-ERBα in microglia versus astrocytes and the limitations of current drugs mean that clinical success will require precise calibration rather than a simple push for maximal receptor activation.
